# The Effectiveness of Interventions on Sustained Childhood Physical Activity: A Systematic Review and Meta-Analysis of Controlled Studies

**DOI:** 10.1371/journal.pone.0132935

**Published:** 2015-07-20

**Authors:** Jamie Sims, Peter Scarborough, Charlie Foster

**Affiliations:** 1 The British Heart Foundation Centre on Population Approaches for Non-Communicable Disease Prevention, Nuffield Department of Population Health, University of Oxford, Oxford, United Kingdom; 2 Department of Sport Development and Management, University of Chichester, Chichester, United Kingdom; National Center of Neurology and Psychiatry, JAPAN

## Abstract

**Background:**

Increased physical activity (PA) has been associated with a reduction in non-communicable disease risk factors and outcomes. However, interventions to increase childhood PA typically produce small to negligible effects. Recent reviews are limited due to lack of post-intervention follow-up measurement. This review aimed to examine measured effects at least six months post-intervention.

**Methods and Findings:**

We searched PubMed, MEDLINE, EMBASE, PsychINFO, ScienceDirect, SportDiscus and Google Scholar between 1^st^ January 1991 and 1^st^ November 2014 for controlled studies reporting six-month post-intervention measurement for children aged 5 to 18 years. 14 studies met inclusion criteria; 12 reported moderate-to-vigorous PA (MVPA) (*n* = 5790) and 10 reported total PA (TPA) (*n* = 4855). We calculated overall effect estimates and 95% CI’s using random effects modelling with inverse variance weighting. Mean difference was calculated for MVPA, with standardised mean difference calculated to TPA due to measurement variation. Meta-regression assessed heterogeneity by continuous level variables. Negligible mean difference in MVPA existed in favour of the intervention group, amounting to 1.47 (95% CI -1.88, 4.82) mins/day compared to controls, while no difference was recorded on TPA. Sub-group analyses revealed males (2.65 mins/day: 95% CI 2.03, 3.27) reported higher levels of MVPA than females (-0.42 mins/day: 95% CI -7.77, 6.94), community settings (2.67 mins/day: 95% CI 2.05, 3.28) were more effective than school settings (1.70 mins/day: 95% CI -4.84, 8.25), and that treatment (4.47 mins/day: 95% CI -0.81, 9.76) demonstrated greater effects than population approaches (1.03 mins/day: 95% CI -2.54, 4.60). Meta-regression revealed no significant differences by factor on pooled effects. Significant heterogeneity existed between studies and potential for small study effects was present.

**Conclusions:**

Improved PA levels subsequent to intervention were not maintained six month post-intervention. A potentially useful avenue of future research is to specifically explore community treatment of high risk individuals.

**Review Registration:**

PROSPERO CRD42014007545

## Introduction

The health consequences of insufficient lifespan physical activity (PA) have been widely reported [1] and strongly associated with increased all-cause mortality [2] and non-communicable diseases [3] such as cardio-vascular disease [4,5], Type II diabetes [6], depression [7], osteoporosis [8] and specific cancers [9], through elevated risk factors such as hypertension, blood glucose, poor cardio-respiratory fitness and adiposity [10–12]. Increased PA is associated with promoting improved energy balance [5,13], bone density [14] and functional movement skills [15].

The inception of many of the above risks have been observed as commencing in childhood [16,17], with a lack of PA leading to impaired childhood health outcomes [18], increased risk factors and subsequent ill-health outcomes in adulthood, and a compromised attitude towards PA [16,19]. PA behaviour tracks ‘reasonably well’ across time, although stability reduces in adolescence and periods of transition [20]. In addition, evidence indicates that PA levels enter a broad decline in later childhood and adolescence [21], resulting in insufficient levels of PA during transition into adulthood [22,23].

The effectiveness of interventions to increase childhood PA has been systematically reviewed; specifically investigating preventative [24,25], treatment-based [26], school-based [27,28] and community-based studies [29] as well as comparative policy reviews [30]. The magnitude of measured effects on levels of PA following intervention has typically been small and, when taking into account consistently high levels of heterogeneity, risk of small sample bias and an over-reliance on self-report measurement, caution is essential when interpreting positive findings. In addition, reviews typically included studies reporting measurement of PA or sedentary behaviour within limited times of day (e.g. school recess, travel time or after-school period), thereby failing to account for potential substitution [31].

These shortfalls were partially addressed in a recent systematic review by Metcalf, Henley and Wilkin [32] who investigated the effectiveness of interventions on levels of childhood PA across 30 controlled studies. Meta-analyses revealed only small-to-negligible effect on levels of Moderate to Vigorous Physical Activity (MVPA) and Total Physical Activity (TPA) immediately following intervention as measured by accelerometry, highlighting the potential for self-report bias in previous reviews and the importance of drawing data from studies specifically reporting whole-day PA [32]. However, with the exception of Lai et al. [28], which focused exclusively on school-based interventions, published reviews provide little detail regarding the maintenance of effects on whole-day PA in children and therefore do not account for the potential effects of habit formation [33] and stage of change [34].

### Aims

Given the shortfall in the literature, the primary objective was to conduct a systematic review to explore the effect of interventions on maintained whole-day childhood PA, including studies that measured physical activity level with either accelerometers or questionnaire. Furthermore, it was necessary to explore sustained effect sizes following a period of at least six months post-intervention.

## Methods

### Search Strategy

The search encompassed PubMed, MEDLINE, EMBASE, PsychINFO, ScienceDirect, SportDiscus and Google Scholar (first 1,000) for studies published between January 1991 and November 2014. Reference lists of included studies and relevant published reviews were hand searched for additional studies. Only English terms were used and only English language studies were included (see Table 1).

**Table 1:** Example Search Criteria for Databases.

child* OR adolescen* OR “young people”
*AND*
“physical activity” OR sport* OR cycl* OR walk* OR “physical education” OR “television view*” OR “tv view*” OR sedentary OR danc* OR “physical inactivity” OR “physical fitness” OR lifestyle OR exercise OR screen time OR “active travel*”OR commut*
*AND*
clinical trial OR control* trial OR random* OR trial OR evaluation OR effect* OR random* sample OR control*

### Study selection

Peer-reviewed studies were included if they utilised a trial design incorporating a non-PA control group, irrespective of whether randomisation was used. No restriction was applied regarding intervention duration, delivery personnel or setting. Inclusion required an intervention(s) targeting PA levels in non-clinical children or adolescents aged between 5–18 years inclusive. Studies must have utilised a measure of MVPA or TPA spanning at least two domains of physical activity obtained either by objective measurement or validated self-report measure. Finally, studies must have presented follow-up measurement data at least six months post-intervention for the same participants measured at baseline and included at least 50% follow-up measurement rate from baseline.

The lead researcher (JS) examined the titles of all studies identified from the initial database results and excluded all publications that were unambiguously irrelevant and duplications. Abstracts were then examined by the lead researcher (JS) and allocated to ‘relevant,’ ‘irrelevant’ and ‘undecided’ groups, with all undecided studies discussed with a second researcher (PS) and resolved through discussion. Full text articles were then accessed and reviewed by the lead researcher (JS), with the second researcher (PS) cross-checking all included studies and the third researcher cross-checking a 10% sample of excluded studies (CF).

### Data extraction and standardisation

We extracted author(s), project title, nation, design, inclusion criteria, randomisation procedure where applicable, intervention and control descriptions, length of follow-up, losses to follow-up and/or drop out, measurement strategy, secondary outcome measures and results. Self-report or objective measurement was recorded, with the specific questionnaire or accelerometer and the length of the measurement period. Participant characteristics were extracted on relative gender percentages, baseline age, baseline BMI or zBMI scores as well as baseline TPA and MVPA levels. Extracted data were entered into an Excel spreadsheet [35] for the purposes of recording and standardisation. Measurement strategy and measurement tools, along with target outcome and quality of reporting, varied considerably between studies necessitating a number of assumptions and transformations.

TPA was measured using either an accelerometer [36–41] or questionnaire [42–44]. MVPA was also measured using accelerometer [36–39,45,46] or questionnaire [43,44,47–50]. To permit meta-analysis on mean differences [51], MVPA effects were transformed into minutes per day. Where Moderate PA and Vigorous PA were presented separately [44] they were combined [52]. Where only Moderate or Vigorous PA was reported [48] this was taken to be sufficiently conceptually similar to MVPA and entered into the meta-analyses as an equivalent main effect. Where MVPA was presented as a percentage of TPA [37,40] the means and standard deviations were multiplied out to provide minutes per day. If effects were given as amount of change [47], this change was added to baseline figures to arrive at a follow-up effect. Where TPA was presented on a log scale [36], means and standard deviations were transformed using standard procedures [52]. Where geometric means were reported [48], it was assumed that these corresponded to the arithmetic means. Where inter-quartile range was reported as the indication of dispersion [48], the quartile points were plotted on an assumed normal distribution and the corresponding standard deviations were entered into the analysis. Where data were presented for separate experimental groups, primarily by gender but also for staggered intervention cohorts, the numbers, means and standard deviations were combined for entry into the meta-analyses [52]. Where specific data was missing from a paper two attempts were made to contact the correspondence author by email.

### Statistical analysis

The group sizes, means and standard deviations were entered into Stata 13 [53], with MVPA and TPA analysed as separate outcomes. The effect sizes of all outcome-relevant studies were combined to provide the overall effect for both MVPA and TPA. The planned outputs were overall effect estimates and 95% confidence intervals using random effects modelling with inverse variance weighting. Random effects was chosen *a priori* as a moderate to high degree of heterogeneity was anticipated between studies [54]. Initial analysis of the papers revealed TPA to have been measured and reported using varied instruments, therefore the effect calculation for TPA used standardised mean difference, while mean difference was calculated for MVPA given the relative suitability of reported measurements to be standardised into mins/day.

### Subgroup analyses

*A priori* subgroup analyses were planned for: participant characteristics (gender, age and cohort size); intervention characteristics (prevention vs. treatment, PA included vs. PA not included, intervention duration and school vs. community setting), and outcome characteristics (objective vs. subjective measurement and post-intervention follow-up delay).

## Results

### Literature search

**Fig 1:**
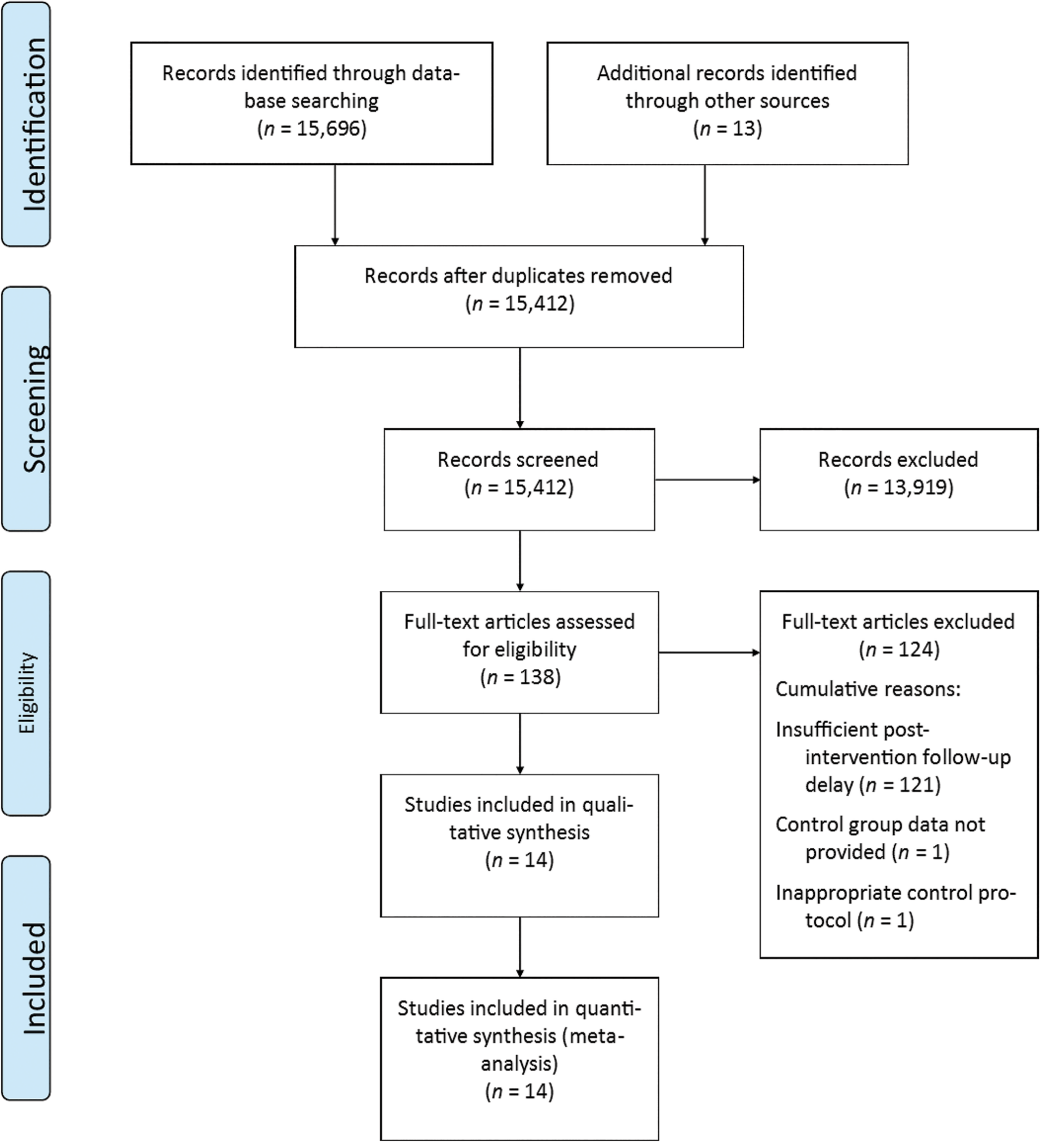
PRISMA Flow Chart Summarising the Study Selection Process.

The searches were conducted and completed in February 2014. The initial search of databases resulted in 15,696 identified studies, with 13 additional studies identified from relevant systematic reviews. Removal of duplicates and analysis of titles then allowed unambiguously ineligible studies to be excluded, leaving a sub-total of 1,493. Scrutiny of abstracts of the remaining studies revealed 138 potentially relevant studies. Full text articles were then reviewed, producing a total of 18 preliminarily studies. Four further studies were excluded at the data extraction stage, leaving 14 studies for the final systematic review. A PRISMA flow-chart [55] of the study selection process is provided in Fig 1.

### Study characteristics

A study-by-study description of the individual characteristics of included studies is provided in Table 2. Seven of the fourteen studies were conducted in the USA [36,38,39,43,47–49], two in Australia [37,45], one in China [41] with one in Hong Kong [44], and one each in Denmark [46], Israel [42] and Portugal [50]. All but one of the studies, therefore, were conducted in high-income nations according to the World Bank economic classifications, with one conducted in the upper middle-income bracket [41]. Overall, five Cluster Randomised Controlled Trials [39,40,44,47,49,50], three Randomised Controlled Trials [36,41,48], two Randomised Prospective Studies [38,42], one Cluster Randomised Prospective Study [43], one Nested Randomised Controlled Trial [37] and one Controlled Longitudinal Trial [46].

**Table 2:** PICOS Summary of Fourteen Included Studies

**Study**	Araujo-Soares et al. [50]
**Methods**	Study Design: Cluster-randomised controlled trial
	Unit of Allocation: Class
	Intervention Period: 12 weeks
	Post-Intervention Follow-Up Period: 3 months and 9 months
	Differences in Baseline Characteristics: Reported
	Unit of Analysis: Child
	Control Strategy: Intention to treat
**Participants**	Control *n*: Baseline = 157; Post-Intervention = 157; 3 Month Follow-Up = 157, 9 Month Follow-Up = 102*
	Intervention *n*: Baseline = 134; Post-Intervention = 134; 3 Month Follow-Up = 134, 9 Month Follow-Up = 90*
	Setting: School
	Recruitment: Not reported
	Location: Northern Portugal
	Percentage of Eligible Population Included: 100%
	Mean Age at Baseline: Intervention = 12.19 ± 1.1, Control = 12.05 ± 0.9
	Baseline Weight Status: Not reported
	Gender: Females = 52.6%
**Interventions**	Manualised delivery by trained psychologist and physical education teacher; children and parent problem-solving groups; action planning and coping planning; behavioural contracts
	Theoretical Grounding: Social Cognitive Theory, Self-Regulation Theory
**Outcomes**	Moderate to Vigorous Physical Activity: Self Report (International Physical Activity Questionnaire)
**Study**	Black et al. [36]
**Methods**	Study Design: Randomised controlled trial
	Unit of Allocation: Individual
	Intervention Period: 3–6 months
	Post-Intervention Follow-Up Period: 18–21 months
	Differences in Baseline Characteristics: Reported
	Unit of Analysis: Child
	Control Strategy: Intention to treat
**Participants**	Control *n*: Baseline = 114; Post-Intervention = 93; 24 Month Follow-Up = 90*
	Intervention *n*: Baseline = 121; Post-Intervention = 91; 9 Month Follow-Up = 89*
	Setting: Community
	Recruitment: Not reported
	Location: Baltimore, USA
	Percentage of Eligible Population Included: 100%
	Mean Age at Baseline: Intervention = 13.3 ± 1.0, Control = 13.3 ± 1.0
	Baseline Weight Status: Intervention = 44.6%, Control = 31.6%
	Gender: Intervention = 51.2%, Control = 47.4%
**Interventions**	Manualised, twelve session intervention for urban, black adolescents; each session incorporates a challenge/goal related to diet/PA; participants engage in PA classes and sample healthy foods; classes and contact is delivered by mentors from a similar background
	Theoretical Grounding: Social Cognitive Theory
**Outcomes**	Anthropometry: zBMI
	Body Composition: Dual-Energy Radiograph Absorptiometry
	Total Physical Activity: Objective (Actiwatch Accelerometer)
	Play-Equivalent Physical Activity: Objective (Actiwatch Accelerometer)
	Diet: Self-Report (Youth Adolescent Food Frequency Questionnaire)
**Study**	Bugge et al. [46]
**Methods**	Study Design: Controlled longitudinal trial
	Unit of Allocation: School
	Intervention Period: 3 years
	Post-Intervention Follow-Up Period: 4 years
	Differenced in Baseline Characteristics: Reported
	Unit of Analysis: Child
	Control Strategy: Match groups for socio-demographic characteristics
**Participants**	Control *n*: Baseline = 225; Post-Intervention = 186; 4 Year Follow-Up = 125*
	Intervention *n*: Baseline = 334; Post-Intervention = 289; 4 Year Follow-Up = 175*
	Setting: School
	Recruitment: Children volunteered from within all schools in two local authority areas
	Location: Copenhagen, Denmark
	Percentage of Eligible Population Included: 100%
	Mean Age at Baseline: Intervention = 6.8 ± 0.4, Control = 6.7 ± 0.4
	Baseline Weight Status (BMI): Intervention = 16.1 ± 1.8, Control = 16.1 ± 1.8
	Gender: 52.45% Female
**Interventions**	Double PE curriculum content
**Outcomes**	Anthropometry: BMI, zBMI, Skinfold, Waist Circumference
	Total Physical Activity: Objective (Actigraph 7164 Accelerometer)
	Moderate to Vigorous Physical Activity: Objective (Actigraph 7164 Accelerometer)
	Cardiovascular Fitness: VO_2_max
**Study**	Chen et al. [41]
**Methods**	Study Design: Randomised controlled trial
	Unit of Allocation: Individual
	Intervention Period: 8 weeks
	Post-Intervention Follow-Up Period: 6 months and 8 months
	Differenced in Baseline Characteristics: Reported
	Unit of Analysis: Child
	Control Strategy: Waiting list
**Participants**	Control *n*: Baseline = 32; Post-Intervention = not reported; 6 Month Follow-Up = 24*
	Intervention *n*: Baseline = 35; Post-Intervention = not reported; 6 Month Follow-Up = not reported; 8 Month Follow-Up = 33*
	Setting: School
	Recruitment: Invited volunteer parents from Chinese-language sources in local area
	Location: San Francisco, USA
	Percentage of Eligible Population Included: 93%
	Mean Age at Baseline: Intervention = 9.14 ± 0.85, Control = 8.78 ± 0.91
	Baseline Weight Status: 81% > 85th percentile
	Gender: 43% Female
**Interventions**	Play-based workshops for children that developed problem-solving towards food choice and physical activity; empower children to develop self-efficacy regarding meal selection and alternatives to sedentary travel and activity; reinforced with family-based meeting to develop social support
	Theoretical Grounding: Social Cognitive Theory
**Outcomes**	Anthropometry: BMI, Wait-to-Hip Ratio
	Total Physical Activity: Objective (Caltrac Accelerometer)
	Diet: Fat, Sugar and Vegetable Consumption
	Dietary Knowledge: Adapted from CATCH Health Behaviour Questionnaire
	Physical Activity Knowledge: Adapted from CATCH Health Behaviour Questionnaire
	Physical Activity Self-Efficacy: Sub-scale from Health Behaviour Questionnaire
**Study**	Cui et al. [49]
**Methods**	Study Design: Cluster randomised controlled trial
	Unit of Allocation: School
	Intervention Period: 4 weeks
	Post-Intervention Follow-Up Period: 6 months
	Differenced in Baseline Characteristics: Reported
	Unit of Analysis: Child
	Control Strategy: Matched School
**Participants**	Control *n*: Baseline = 371; Post-Intervention = not reported; 6 Month Follow-Up = 336*
	Intervention *n*: Baseline = 358; Post-Intervention = not reported; 6 Month Follow-Up = 346*
	Setting: School
	Recruitment: Not reported
	Location: Beijing, China
	Percentage of Eligible Population Included: 100%
	Mean Age at Baseline: Intervention = 12.7 ±, Control = ± 12.7
	Baseline Weight Status: Intervention = 36.2%, Control = 29.4% Overweight or Obese
	Gender: Female = 48%
**Interventions**	Four-component intervention adapted from Shah, van der Sluijs, Lagleva, Pesle, Lim et al. [60], comprising food choice, physical activity, sedentary behaviour and carbonated drink consumption; achieved through a peer-led information provision and goal setting programme
	Theoretical Grounding: Social Cognitive Theory
**Outcomes**	Moderate to Vigorous Physical Activity: Validated 7-Day Physical Activity Questionnaire [61]
	Physiological: Height, Weight and BMI
**Study**	Dewar et al. [40]
**Methods**	Study Design: Cluster randomised controlled trial
	Unit of Allocation: School
	Intervention Period: 12 months
	Post-Intervention Follow-Up Period: 12 months
	Differences in Baseline Characteristics: Reported
	Unit of Analysis: Child
	Control Strategy: Waiting list
**Participants**	Control *n*: Baseline = 179; Post-Intervention = 153; 12 Month Follow-Up = 153*
	Intervention *n*: Baseline = 178; Post-Intervention = 141; 6 Month Follow-Up = 141*
	Setting: School
	Recruitment: Invitation to schools randomly selected from within area
	Location: NSW, Australia
	Percentage of Eligible Population Included: 100%
	Mean Age at Baseline: 13.2 ± 0.5
	Baseline Weight Status: 42.9% Overweight or Obese
	Gender: Female = 100%
**Interventions**	Enhanced school sport and lunchtime PA sessions, nutrition workshops; mediators targeted with additional PA sessions, seminars, student handbooks, parent newsletters & text messages
	Theoretical Grounding: Social Cognitive Theory
**Outcomes**	Moderate to Vigorous Physical Activity: Objective (Actigraph Accelerometer)
	Sedentary Behaviour: Objective (Accelerometer); Self-Report (Adolescent Sedentary Activity Questionnaire)
	Psychosocial Variables: Self-Report scale constructed for measurement
**Study**	Hovell et al. [48]
**Methods**	Study Design: Randomised controlled trial
	Unit of Allocation: Individual
	Intervention Period: 8 weeks
	Post-Intervention Follow-Up Period: 6 months & 10 months
	Differences in Baseline Characteristics: Reported and adjusted; male and female data analysed separately due to significant difference at baseline
	Unit of Analysis: Child
	Control Strategy: Child safety intervention replaced diet and PA intervention
**Participants**	Control *n*: Baseline = 60; Post-Intervention = 49; 6 Month Follow-Up = 49, 10 Month Follow-Up = 44*
	Intervention *n*: Baseline = 78; Post-Intervention = 68; 6 Month Follow-Up = 66, 10 Month Follow-Up = 62*
	Setting: Outpatient
	Recruitment: Sequential over three years, recruited from advertisements and agency referrals
	Location: San Diego, USA
	Percentage of Eligible Population Included: 100%
	Mean Age at Baseline: Cohort = 11.48 ± 0.96
	Baseline Weight Status: Not reported
	Gender: Females = 58.1% Female
**Interventions**	Outpatient-based weekly sessions for 90 minutes with separate parent and child classes; parent training classes focussed on behaviour management, bone health, diet and PA; telephone support for parents throughout the intervention period to support behaviour management; child classes focussed on high impact PA participation and importance of calcium rich food
	Theoretical Grounding: None reported
**Outcomes**	Diet: Calcium intake & total energy intake using 24 hour recall conducted by telephone
	Moderate to Vigorous Physical Activity: 24 hour recall of specific high impact activities conducted by telephone
	Physiological: Bone mineral density, bone mineral content & body composition
**Study**	Jago et al. [39]
**Methods**	Study Design: Cluster randomised controlled trial
	Unit of Allocation: Scout troop
	Intervention Period: 9 weeks
	Post-Intervention Follow-Up Period: 6 months
	Differenced in Baseline Characteristics: Reported
	Unit of Analysis: Child
	Control Strategy: Non-PA Fruit & Vegetables Guidance
**Participants**	Control *n*: Baseline = 233; 6 Month Follow-Up = 233*
	Intervention *n*: Baseline = 240; 6 Month Follow-Up = 240*
	Setting: Scout Troop
	Recruitment: Not reported
	Location: Houston, USA
	Percentage of Eligible Population Included: 100%
	Mean Age at Baseline: Cohort = 13 ± 0.1
	Baseline Weight Status: Not reported
	Gender: Females = 0% Female
**Interventions**	Badge-based engagement in PA goal-setting intervention within Scout Troops; badge intervention included goal setting, scout booklet & physical activity sessions; web-based tracking of behaviour and goal-setting, participants logged on twice weekly
	Theoretical Grounding: None reported
**Outcomes**	Physiological: Height, Weight, BMI, Triceps Skinfold
	Total and Moderate to Vigorous Physical Activity: Objective (MTI Accelerometer)
**Study**	McManus et al. [44]
**Methods**	Study Design: Cluster randomised controlled trial
	Unit of Allocation: School
	Intervention Period: 6 weeks
	Post-Intervention Follow-Up Period: 6 months
	Differences in Baseline Characteristics: Reported
	Unit of Analysis: Child
	Control Strategy: As intervention without healthy heart education or extra PA
**Participants**	Control *n*: Baseline = 69; 6 Month Follow-Up = 66*
	Intervention *n*: Baseline = 67 6 Month Follow-Up = 63*
	Setting: School
	Recruitment: Random selection from local school list
	Location: Hong Kong, Republic of China
	Percentage of Eligible Population Included: 100%
	Mean Age at Baseline: Cohort = 10.44 ± 0.85
	Baseline Weight Status: Not reported
	Gender: Females = 50% Female
**Interventions**	Healthy heart training and additional PE sessions; two-week educational programme incorporated within PE classes; explicit heart-rate monitors and goal setting process
	Theoretical Grounding: Health Belief Model; Social Cognitive Theory; Diffusion of Innovation Theory
**Outcomes**	Moderate to Vigorous Physical Activity: Continuous heart rate telemetry
	Attraction to PA: Self-Report (Children's Attraction to Physical Activity Scale)
	Physiological: Weight, height, hip circumference, resting and exercise cardiopulmonary rate
**Study**	Nader et al. [43]
**Methods**	Study Design: Cluster randomised prospective study
	Unit of Allocation: School
	Intervention Period: 3 Years
	Post-Intervention Follow-Up Period: 3 Years
	Differences in Baseline Characteristics: Reported (not for PA)
	Unit of Analysis: Child
	Control Strategy: Outpatient monitoring and ambulatory/dietary given, PA levels recommended
**Participants**	Control *n*: Baseline = 2117 (full cohort); 3 Year Follow-Up = 1400* (PA measurement sub-section)
	Intervention *n*: Baseline = 2989 (full cohort); 3 Year Follow-Up = 1996* (PA measurement sub-section)
	Note—No PA measurement occurred at baseline
	Setting: School
	Recruitment: All schools from 4 geographical centres invited to participate, 96 schools included
	Location: San Diego, Minnesota, Austin & New Orleans, USA
	Percentage of Eligible Population Included: 100%
	Mean Age at Baseline: Cohort = 8.76
	Baseline Weight Status: Not reported
	Gender: Females = 49% Female
**Interventions**	Comprehensive programme of curricular and extra-curricular PA and Nutrition intervention, comprising: Eat Smart—School meals provided with lower fat and sodium content; CATCH PE—Trained PE staff to deliver enjoyable and engaging MVPA participation; numerous classroom based problem-solving and content aimed at inspiring greater levels of PA; 19 Home-delivery packets encouraging whole-family activity and family fun nights
	Theoretical Grounding: None reported
**Outcomes**	Total and Moderate to Vigorous Physical Activity: Self-Administered Physical Activity Checklist (SAPAC)
	Nutrition: 24-Hour Dietary Recall; Food Checklist
	Health Behaviour: Health Behaviour Survey (HBS)
	Physiological: Blood Cholesterol; HDL Cholesterol; Apolipoprotein B Levels; Height, Weight, Skinfold Thickness, Blood Pressure
**Study**	Nemet et al. [42]
**Methods**	Study Design: Randomised prospective study
	Unit of Allocation: Individual
	Intervention Period: 3 months
	Post-Intervention Follow-Up Period: 9 months
	Differences in Baseline Characteristics: Reported
	Unit of Analysis: Child
	Control Strategy: Outpatient monitoring and ambulatory/dietary given, PA levels recommended
**Participants**	Control *n*: Baseline = 22; 9 Month Follow-Up = 20*
	Intervention *n*: Baseline = 24; 9 Month Follow-Up = 20*
	Setting: Outpatient clinic
	Recruitment: Self-referral
	Location: Tel Aviv, Israel
	Percentage of Eligible Population Included: 100%
	Mean Age at Baseline: Control = 11.3 ± 2.8, Intervention = 10.9 ± 1.9
	Baseline Weight Status: 100% Obese
	Gender: Females = 45% Female
**Interventions**	Dietary intervention for parents and children focusing on nutritional education; exercise programme occurred twice weekly for children focusing on a variety of activities, including walking
	Theoretical Grounding: None reported
**Outcomes**	Anthropomorphic: Height, Weight, BMI, Triceps & Subscapular Skinfolds
	Nutrition: Self-Report (2-Day Food Intake Diary)
	Total Physical Activity: Self-Report [62]
	Fitness: Progressive Treadmill Test
	Physiological: Triglycerides, Cholesterol, High-Density Lipoprotein
**Study**	Roemmich et al. [38]
**Methods**	Study Design: Randomised prospective study
	Unit of Allocation: Individual
	Intervention Period: 4 months
	Post-Intervention Follow-Up Period: 8 months
	Differences in Baseline Characteristics: Reported
	Unit of Analysis: Child
	Control Strategy: No intervention group, accelerometer display turned off, limited screen time
**Participants**	Control *n*: Baseline = 21; 8 Month Follow-Up = 21*
	Intervention *n*: Baseline = 20; 8 Month Follow-Up = 20*
	Setting: Community
	Recruitment: Stratified random invitation to families, only one child per household
	Location: New York, USA
	Percentage of Eligible Population Included: 100%
	Mean Age at Baseline: Control Males = 11.3 ± 1.8, Females = 10.5 ± 1.6; Intervention Males = 10.5 ± 1.5, Females = 11.2 ± 1.1
	Baseline Weight Status: 0% Overweight
	Gender: Females = 50% Female
**Interventions**	Family-based intervention involving conditional screen time; problem solving opportunities to participate in PA; provision of accelerometer with visible display in order to 'purchase' screen-based activities
	Theoretical Grounding: None reported
**Outcomes**	Anthropomorphic: Height, Weight, z-BMI
	Total and Moderate to Vigorous Physical Activity: Objective (BioTrainer-Pro Accelerometry)
	Sedentary Time: Self-Report (Habit Book)
**Study**	Wake et al. [37]
**Methods**	Study Design: Nested randomised controlled trial
	Unit of Allocation: Individual
	Intervention Period: 12 weeks
	Post-Intervention Follow-Up Period: 9 months
	Differences in Baseline Characteristics: Reported
	Unit of Analysis: Child/Family
	Control Strategy: Non-intervention group within GP practices
**Participants**	Control *n*: Baseline = 119; 3 Month Follow-Up = 109; 9 Month Follow-Up = 91*
	Intervention *n*: Baseline = 139; 3 Month Follow-Up = 122; 9 Month Follow-Up = 110*
	Setting: Community/Primary Care
	Recruitment: Participants invited into over-arching survey by practice staff, random sample invited to join trial
	Location: Melbourne, Australia
	Percentage of Eligible Population Included: 100%
	Mean Age at Baseline: Control = 7.6 ± 1.4; Intervention = 7.4 00B1 1.4
	Baseline Weight Status: 100% Overweight
	Gender: Females = 60% Female
**Interventions**	Incorporating existing intervention into current study from LEAP trial, comprising brief solution-focused conducted by GP; a folder is provided to the child to consolidate therapy sessions, consisting of healthy lifestyle goals related to healthy family eating, physical activity, sedentary time, water consumption and lower fat food options
	Theoretical Grounding: None reported
**Outcomes**	Total and Moderate to Vigorous Physical Activity: Objective (Actical Mini-Mitter Accelerometer), 4-Day Parent Report (non-validated)
	Physiological: Height, Weight, BMI, Waist Circumference, Maternal and Paternal BMI
	Nutrition: 4-Day Food Report Diary; Parental Report
	Health Status: Pediatric quality of life inventory (PedsQL 4.0)
	Psychological: Body Dissatisfaction (Body Figure Perception Questionnaire); Physical Appearance and Self-Worth (Harter's Perceived Competence Scale)
**Study**	Wright et al. [47]
**Methods**	Study Design: Cluster randomised controlled trial
	Unit of Allocation: School
	Intervention Period: 6 weeks
	Post-Intervention Follow-Up Period: 12 months
	Differences in Baseline Characteristics: Reported
	Unit of Analysis: School
	Control Strategy: Standard education
**Participants**	Control *n*: Baseline = 130; 12 Month Follow-Up = 90*
	Intervention *n*: Baseline = 121; 12 Month Follow-Up = 91*
	Setting: School
	Recruitment: Drawn specifically from low SES schools
	Location: Los Angeles, USA
	Percentage of Eligible Population Included: 82%
	Mean Age at Baseline: Control = 8.3 ± 1.1; Intervention = 9.0 ± 1.6
	Baseline Weight Status: Not reported
	Gender: Females = 50% Female
**Interventions**	The Kids N Fitness [63] comprises nurse-led group meetings focusing on engaging in physical activity, nutrition education/behaviour modification and family support; the meetings contained a participation component, an educational component and a goal-setting and problem solving component
	Theoretical Grounding: None reported
**Outcomes**	Moderate to Vigorous Physical Activity: Self-Report Child and Adolescent Trial for Cardiovascular Health (CATCH) School Physical Activity and Nutrition (SPAN) Student Questionnaire
	Anthropomorphic: Height, Weight, BMI, zBMI, Resting Blood Pressure, Waist Circumference

* Data entered into meta-analysis

#### Participant characteristics

The number of participants ranged between the smallest study of 41 [42] and the largest of 3,714 [43]. The median sample size was 255, with a total number of 7883 participants. Overall, 51.27% of the participants were female (range: 0% to 100%, median 49.5%) with one study recruiting only females [40] and one using only males [39]. Only two studies reported gender-specific results [46,48]. Mean baseline age was 10.67 (± 1.91), with eight studies targeting participants of UK primary education age [37,38,41–44,46,47] and six studies targeting UK secondary education age participants [36,39,40,48–50]. Three studies were treatment orientated [37,42,47], recruiting specifically overweight or obese participants, with the remainder being promotional or preventative.

#### Intervention characteristics

Studies provided extra physical education classes in curriculum time [43,46,50], PA delivery outside curriculum time [39,42], counselling [37], goal-setting sessions [50], incentive-based interventions [38] and peer-modelling [36,49] either singularly or in combination. Five studies [36,40,41,49,50] reported explicitly grounding an intervention strategy in Social Cognitive Theory [56]. Seven studies [40,43,44,46,47,49,50] involved a school setting within the intervention delivery, and seven in a community setting; three in a standard community approach [36,38,41,48] two in a primary care setting [37,42], one as part of a scout group [39]. Intervention duration lasted between six weeks [44] and three years [43,46], with a median of three months.

#### Control characteristics

None of the control groups included a PA component, excepting those comparisons which were made between additional PA and ‘normal practice’ in which case the participants completed standard physical education classes within curriculum time. Differences in characteristics between baseline and intervention groups were reported in all cases, with no comparisons deemed to be at high risk of bias. Studies using a cluster-design reported methods to ensure groups were comparable at baseline.

#### Outcome characteristics

Twelve studies reported MVPA [36–40,43,44,46–50] and ten studies [36–44,46] reported TPA, seven using objective [36–41,46] and seven using self-report measures [42–44,47–50]. The self-report measures included standardised questionnaires, 24-hour recall and participation tick-sheets. Of the fourteen studies eight reported both TPA and MVPA [36–40,43,44,46]. Follow-up measurement ranged from six months [39,41,44,49] to four years [46], with a median delay of nine months post-intervention. Loss to follow up ranged from 0% [38,39] to 48% [48] with the median loss being 21%.

### Study quality

**Fig 2:**
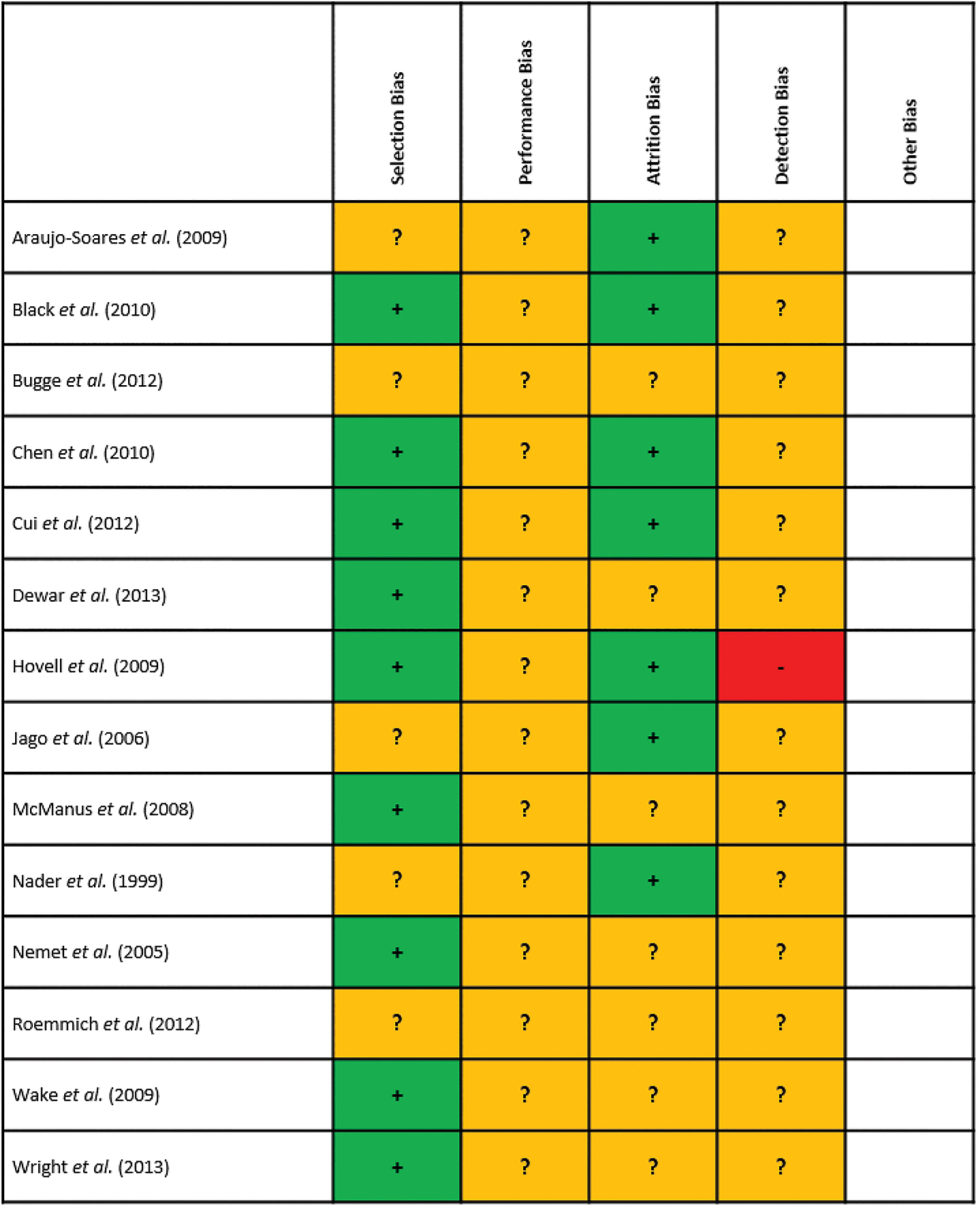
Overall Assessed Risk of Bias within the 14 Included Studies.

**Fig 3:**
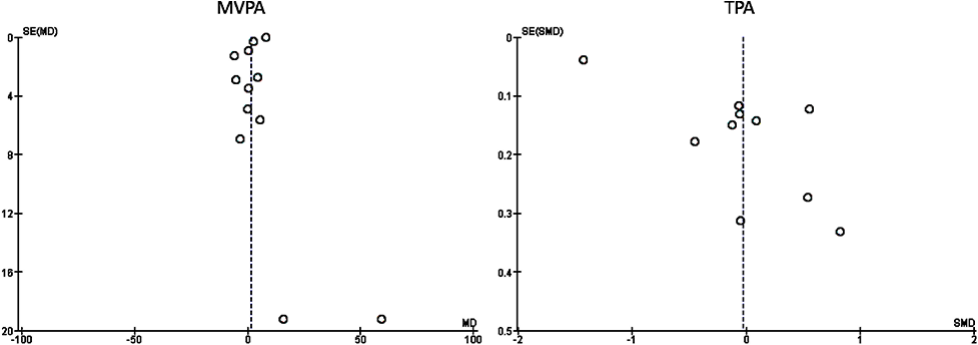
Funnel Plots Showing the Observed Effects for the 12 studies reporting MVPA (left) and the 10 studies reporting TPA (right).

Quality was assessed using the Methodology Checklist for Randomised Controlled Trials [57]. Overall there was a high number of ‘uncertain’ verdicts against the papers, potentially indicating the reporting of relevant information within the published articles was more pertinent than the actual methodological quality of the studies (Fig 2). Participants lost to follow-up ranged from 0% to 50%, with studies reporting analyses of attrition characteristics. Eight of the nine studies utilising cluster-randomised design reported appropriate statistical techniques by which to account for clustering within the aggregate outcomes. A visual inspection of funnel plots for both outcomes suggested the possibility of small-study effect (Fig 3).

### Overall effect estimates

**Fig 4:**
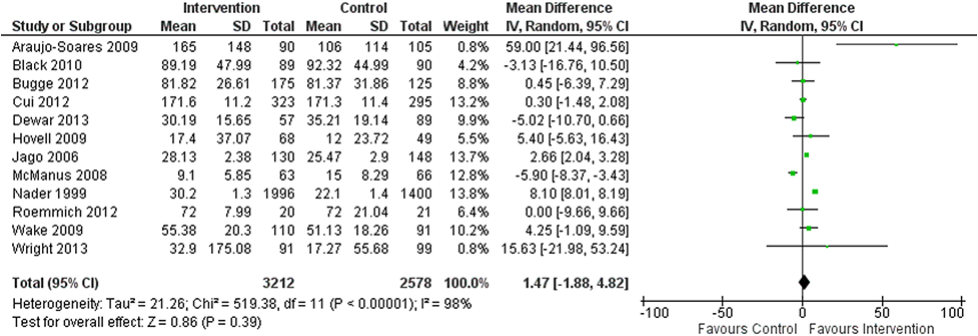
Forest Plot of Mean Difference in Change in MVPA between Intervention (n = 3212) and Control (n = 2578). Groups across the 12 included studies reporting MVPA data.

**Fig 5:**
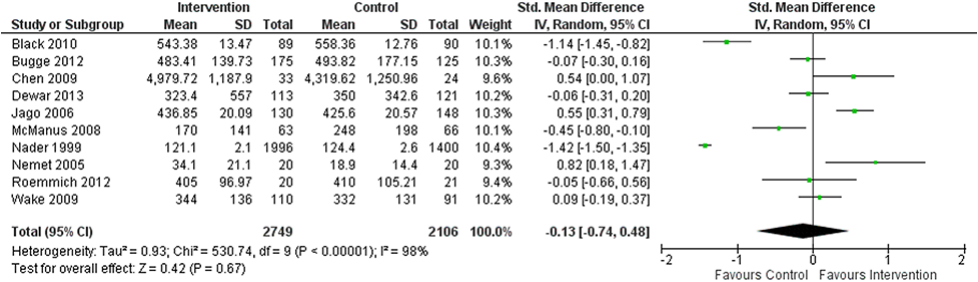
Forest Plot Showing the Standardised Mean Difference in Change in TPA between Intervention (n = 2749) and Control (n = 2106). Groups across the 10 included studies reporting TPA data.

The collated results from twelve included studies showed weak evidence for a small increase in MVPA in favour of the intervention group with a mean difference of 1.47 minutes per day (95% CI -1.88, 4.82; *p* = 0.39) (Fig 4). For the ten studies reporting TPA the analysis showed no difference between the pooled effects of the intervention and those for the control group, with a standardised mean difference of -0.13 (95% CI -0.74, 0.48; *p* = 0.67) (Fig 5). Of most successful studies Araujo-Soares et al. [50] reported a mean difference 59 mins/day (95% CI 21.44, 96.56; *p* = 0.002) of additional MVPA and Nemet et al. [42] reported a standardised mean difference of 0.82 (95% CI 0.18, 1.47, *p* = 0.01). I^2^ values of 98% for MVPA (*p* < 0.001) and TPA (*p* < 0.001) revealed high levels of statistical heterogeneity between studies within both outcomes and a consequential requirement for caution with interpreting the results.

### Subgroup analysis

There were no significant differences in outcomes across the majority of study level characteristics, summarised in Table 3. Individual meta-regressions of MVPA and TPA by continuous level covariates confirmed the lack of statistical significance. Exceptions included male participants showing a mean difference (*p* < 0.001) in MVPA between intervention and control groups at post-intervention follow-up measurement, approximately equivalent to 2.65 mins/day and community-based interventions showed an effect (*p* < 0.01). However Jago et al. [39], due to a significant effect and tight confidence intervals, accounted for the majority of the weighting within the pooled effects on these sub-groups; removal of this paper from the sub-group analysis produced non-significant results. The relative success of community-based interventions may be due in part to small study effect (Fig 3), in which systematic bias is introduced into meta-analyses due to publications bias against studies with small cohorts with non-significant effects [52]. Lastly, the treatment-based subgroup [37,47] that approached significance (*p* < 0.10) for MVPA, and also the Nemet et al. [42] study, were all conducted in community settings, potentially indicating that treatment and community approaches may cluster to promote sustained PA.

**Table 3:** Summary of Effects and p Values for MVPA and TPA Outcomes by Sub-Group across 14 Studies.

Groups	Characteristic	Subgroup	MVPA Effect Estimate		TPA Effect Estimate	
			(mean difference in mins/day)	95% Confidence Intervals	(standardised mean difference)	95% Confidence Intervals
		Overall	1.47	-1.88, 4.82	-0.13	-0.74, 0.48
**Participant Characteristics**	**Gender**	Males	2.65**	2.03, 3.27	0.24	-0.39, 0.87
		Females	-0.42	-7.77, 6.94	-0.08	-0.28, 0.12
	**Educational Age**	Primary	1.96	-5.75, 9.67	-0.10	-0.84, 0.64
		Secondary	0.86	-2.31, 4.04	-0.21	-1.12, 0.71
	**Cohort Size**	< = 275 Participants	0.10	-5.76, 5.96	-0.09	-0.79, 0.62
		>275 Participants	2.49	-1.47, 6.44	-0.18	-1.11, 0.74
**Intervention Characteristics**	**Intervention Approach**	Prevention	1.03	-2.54, 4.60	-0.27	-0.95, 0.41
		Treatment	4.47*	-0.81, 9.76	0.40	-0.31, 1.11
	**Intervention Strategy**	PA Included	1.57	-1.89, 5.04	-0.17	-0.86, 0.52
		PA Not Included	0.00	-9.66, 9.66	0.07	-0.19, 0.32
	**Intervention Setting**	School	1.70	-4.84, 8.25	-0.50	-1.37, 0.36
		Community	2.67**	2.05, 3.28	0.12	-0.49, 0.73
**Outcome Characteristics**	**Measurement Strategy**	Objective	1.03	-1.80, 3.85	-0.03	-0.44, 0.38
		Self-Report	4.04	-3.26, 11.34	-0.39	-1.50, 0.71
	**Intervention Duration**	<4 Months	0.89	-2.80, 4.59	0.05	-0.54, 0.64
		> = 4 Months	1.75	-5.66, 9.16	-0.41	-1.34, 0.52
	**Follow-Up Duration**	< = 9 Months	0.69	-3.10, 4.48	0.23†	-0.15, 0.60
		>9 Months	1.99	-4.77, 8.75	-0.67†	-1.49, 0.15

** *p* < 0.05

* *p* < 0.10 for within group effect

† *p* < 0.05 for between group effect

## Discussion

There was a statistically non-significant (*p* = 0.39) mean difference in favour of intervention, approximating to a mean improvement of 1.47 minutes per day of MPVA compared to controls, although this figure is well below the sensitivity threshold of the utilised measurement tools. This result falls well short of the recommended improvements of PA for children [1] and is unlikely to be clinically significant even if maintained over time. There was no statistically significant (*p* = 0.87) difference in standardised mean difference of TPA. In the case of Cui et al. [49], the control group was assessed at six months post-baseline, rather than post-intervention, although one-study removed sensitivity analyses revealed no meaningful change to overall or sub-group effects. A similar analysis for Hovell et al. [48] was conducted given this papers reporting of geometric, rather than arithmetic, means with no differences found on the sub-group effects.

In PA studies it is typically not possible to blind participants or instructors to allocation, opening a potential source of bias into the delivery [58]. In addition, the measurement was often conducted by researchers not blinded to allocation [59], although sub-group analysis revealed no difference between self-report and objective measures for MVPA or TPA. Levels of heterogeneity apparent between studies that used self-report was consistently high across both outcomes (MVPA I^2^ = 98%; TPA I^2^ = 97%), potentially compromising the sensitivity of this measurement strategy to reliably demarcate significant from non-significant results in small studies.

Negligible effect on the main outcomes was consistent with Metcalf et al. [32], who conducted a meta-analysis on 30 studies measured by accelerometer immediately post-intervention, with Dobbins et al. [27], who reviewed 44 studies specifically regarding school-based interventions, and with Kamath et al. [24], who reviewed 18 studies on PA levels following interventions within a wider review into prevention of childhood obesity. Also concordant with Metcalf et al. [32], findings indicated that intervention duration was not associated with increased PA levels at follow-up, with an emergent trend that favoured studies implemented in a community setting, those that used a treatment approach and those with smaller cohort sizes, potentially implicating a cluster of factors associated with greater intervention success. However, it was not possible to distinguish between specific factors or rule out small study bias. No evidence for harmful effects of intervention on PA was indicated.

The strengths of the current review lay in the specificity and uniqueness of the inclusion criteria regarding methodological approach, requiring follow-up measurement to have occurred at least six months post-intervention, presenting a meaningful analysis to the literature. Limitations included the relatively small number of included studies which left subgroups underpowered within the analyses. In addition, the use of exclusively English language publications introduced a potential for English bias. While the use of a single researcher to conduct the primary identification and extraction procedure may be seen to constitute a weakness the specificity of the inclusion criteria, particularly the clear requirement for a six-month post-intervention follow-up measurement, reduced the likelihood of selection error.

This review reinforced previous evidence that PA interventions have little measured effect on TPA or MVPA levels in children, either immediately post-intervention or at six-month follow-up. The possibility remains that the included studies, plus PA interventions in general, were ineffective due to insufficiencies in intensity, duration, delivery quality, theoretical grounding and implementation or measurement sensitivity. Although the benefits of PA in childhood are intuitive, evidence has yet to support this viewpoint and resources may be better invested in alternative approaches to achieve positive effects. In terms of recommendations for future research, we suggest the inclusion of a rigorously implemented and reported follow-up measurement stage is incorporated into the method, as further publication of pre-post studies will not meaningfully add to the existing literature.

At the time of writing no publication had specifically investigated the maintenance of PA levels at follow-up; this represented an important gap in knowledge addressed by the current review. Sub-group analysis revealed a potential area of promise with the utilisation of PA intervention to treat of high risk children and warrants further investigation. The challenge remains to ensure that high methodological quality, particularly regarding measurement tools, is adhered to in future studies in order to build a meaningful evidence base.

## Supporting Information

S1 PRISMA Checklist Completed PRISMA Checklist. (DOCX)

S1 File Extraction Data from Fourteen Included Studies. (XLSX)
